# Metagenomic Profiling of Barn Owl ( *Tyto javanica javanica* ) Regurgitated Pellets Reveals Spatially Structured Environmental Microbiota in Paddy Agroecosystems

**DOI:** 10.1002/ece3.74429

**Published:** 2026-09-23

**Authors:** Kamarul Zaman Zarkasi, Mohd Hasif Ahmad Kamal, Muhammad Deanni Ronidean, Muhammad Syafiq Mohd Zaludin, Mohammad Feizal Daud, Susiana Purwantisari, Amir Hamzah Ahmad Ghazali, Hasber Salim

**Affiliations:** ^1^ Microbial Ecology and Bacteriology Laboratory, School of Biological Sciences Universiti Sains Malaysia Minden Pulau Pinang Malaysia; ^2^ Barn Owl and Rodent Research Group (BORG), Universiti Sains Malaysia Minden Pulau Pinang Malaysia; ^3^ Faculty of Plantation and Agrotechnology Universiti Teknologi MARA Malacca Branch, Jasin Campus Merlimau Malacca Malaysia; ^4^ Field Crops Production and Crop Quality Product Research Group, Faculty of Plantation and Agrotechnology Universiti Teknologi MARA Malacca Branch, Jasin Campus Merlimau Malacca Malaysia; ^5^ Department of Biology, Faculty of Science and Mathematics Universitas Diponegoro Semarang Central Java Indonesia

**Keywords:** bacterial community, barn owl, diversity, metagenomic, paddy fields

## Abstract

The tropical barn owl ( *Tyto javanica javanica* ) is a major biocontrol agent against rodents in paddy fields. While raptors are frequently monitored for zoonotic diseases, they may also facilitate beneficial microbial redistribution. This study evaluated bacterial communities in barn owl regurgitated pellets to assess their role as environmental biosensors and vectors for microbe dispersal. We collected 90 fresh pellets from three paddy field locations during the growing season. Samples underwent bacterial enumeration and metagenomic sequencing of the 16S rRNA V3‐V4 domains via the Illumina MiSeq platform. Results revealed significant spatial variation in bacterial communities across locations (PERMANOVA, *F*(2, 87) = 3.14, *p* < 0.05). Firmicutes (28.1%–63.2%) and Proteobacteria (17.7%–45.6%) dominated all sites. Notably, *Sporosarcina* sp., known for eco‐friendly biocementation and soil enhancement, was highly abundant across all locations (up to 40.6%). The high abundance of *Escherichia* sp. at the AV site (32.1%) highlighted the pellet's ability to capture transient environmental and prey‐derived coliforms. Ultimately, these pellets act as critical environmental proxies capturing localized microbiota. This supports the perspective that barn owls contribute significantly more to microbial ecological redistribution than to disease spread, offering valuable agricultural ecological services.

## Graphical abstract

Metagenomic analysis of regurgitated pellets reveals that tropical barn owls act as critical environmental biosensors in paddy fields. Findings demonstrate these owls contribute significantly more to the beneficial redistribution of environmental microbes than to spreading zoonotic diseases, providing valuable ecological services to agroecosystems.

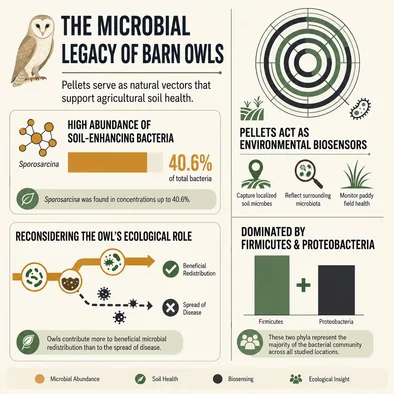

## 1 Introduction

The tropical barn owl ( *Tyto javanica javanica* ) is a nocturnal raptor characterized by a suite of distinct behavioral and morphological adaptations (Noh et al. 2023), including exceptional low‐light visual acuity, highly sensitive auditory capabilities, and a specialized cervical vertebral structure. Geographically, the species exhibits a broad distribution across diverse habitats worldwide, ranging from natural landscapes to urbanized settings, though it is notably absent from extreme environments such as polar regions and hyper‐arid deserts (Andrade et al. 2016; Séchaud et al. 2021). Within agricultural ecosystems, particularly paddy fields, the barn owl functions as a prominent biological control agent that is significantly mitigating rodent infestations and minimizing associated crop damage (Zainal Abidin et al. 2022).

Ubiquitous to all living organisms, microbial communities play an ecologically significant role, particularly within the gastrointestinal tract. In avian species, the composition and dynamics of these bacterial assemblages are shaped by a complex interplay of autochthonous and allochthonous factors (Faniyi et al. 2019; Bindari and Gerber 2022). The key determinants influencing avian microbiota include host genetics, age, environmental exposure, and most notably, dietary composition (Grond et al. 2019; Noh et al. 2023). Diet acts as a pivotal modulator of the bacterial composition within the host, fundamentally influencing digestive processes, gut pH, and overall microbial diversity (Faniyi et al. 2019; Bindari and Gerber 2022).

Given their predatory diet and frequent interaction with agricultural environments, barn owls harbor a complex microbiota that includes both beneficial microorganisms and potentially pathogenic bacteria (Lucas and Heeb 2005; Sun et al. 2022). Previous research has identified potentially harmful microbes in various other avian species, which raises critical concerns regarding the capacity of wild raptors to carry human pathogens and serve as vectors for zoonotic diseases in farming communities (Silvanose et al. 2001; Waite and Taylor 2015; Sun et al. 2022). Conversely, avian hosts may also act as reservoirs for beneficial microbes that possess functional potential for biofertilization, bioremediation, or environmental biocontrol (Waite and Taylor 2014; Grond et al. 2019; Abdullahi et al. 2021; Sun et al. 2022). Despite the recognized reliance on barn owls for integrated pest management, there remains a critical gap in understanding the precise bacterial diversity associated with *T. javanica javanica* in tropical agroecosystems.

Consequently, this study aimed to characterize the composition and diversity of bacterial communities associated with barn owls inhabiting paddy fields during the active paddy growing season. By elucidating this microbial profile, the research sought to understand the potential for the spread and infection of harmful microbes harbored by these birds. Ultimately, evaluating these bacterial dynamics was vital for preventing infectious diseases among farmers while concurrently assessing the owl's broader role in the ecological redistribution of environmentally beneficial microorganisms within their agricultural habitats.

## 2 Materials and Methods

### 2.1 Sample Collection

Samples were collected during the paddy growing season from three distinct paddy field locations in Pulau Pinang, Malaysia: Permatang Tinggi (AV; 5.545551° N, 100.436565° E), Kampong Bumbung Lima (BL; 5.552876° N, 100.438796° E), and Paya Keladi (JK; 5.539965° N, 100.452338° E). A total of 90 wild barn owl regurgitated pellets were obtained over a period of 12 weeks between April and June 2024, comprising 30 pellets from each of the three sampling sites. Pellets were selected based on fresh physical characteristics, specifically being soft, black, moist, and emitting a strong odor. Collections were performed from unoccupied man‐made nests located within the paddy fields to avoid direct contact with or disturbance to the birds. Sampled pellets were placed into tubes, stored in an icebox for transport to the laboratory, and subsequently preserved in a −20°C freezer.

### 2.2 Bacterial Enumeration

The collected pellets were mixed, crushed, and dissolved. The samples were suspended in 200 mL of distilled water, from which 10 mL of the resulting solution was transferred into a fresh Falcon tube and agitated on a shaker for homogenization. Serial dilutions were prepared, and the samples were plated onto three distinct agar media: nutrient agar (NA), eosin methylene blue agar (EMB), and thiosulphate citrate bile salts sucrose agar (TCBS). The plates were incubated at 30°C for 24 h. Following incubation, the resulting colonies were observed, counted, and recorded to determine the average total viable count (TVC) for the bacterial populations per sampling locations.

### 2.3 Genomic DNA Extraction and 16S rRNA Gene Sequencing Genomic

DNA was extracted from the samples utilizing the SPINeasy DNA Kit for Feces/Soil (Cat No: 6547050, MP Biomedicals) in accordance with the manufacturer's standard protocols. Sequencing targeted the V3‐V4 region of the 16S rRNA gene (Zarkasi et al. 2019). Amplification utilized primers equipped with 12 bp barcode tags: 27F (5′‐AGAGTTTGATCMTGGCTCAG‐3′) and 907R (5′‐CCGTCAATTCMTTTRAGTTT‐3′) (Go et al. 2024). Next‐generation sequencing was executed on the Illumina MiSeq platform, with library normalization and pooling conducted following Illumina's official protocol.

### 2.4 Bioinformatics and Statistical Analysis

Raw of sequencing data were subjected to DADA2 analysis (an open‐source pipeline for modeling and correcting Illumina‐sequenced amplicon errors) by the sequencing provider. Paired‐end reads were converted to Fastq format, assessed for quality, and stripped of primers and adapters. Data preprocessing for this pipeline involved trimming, filtering, and denoising to generate an amplicon sequence variant (ASV) table detailing unique sequence variants and their respective abundances (Mokhtar et al. 2023). Chimera screening and taxonomic assignments were conducted using the SILVA database, while phylogenetic trees were constructed using MUSCLE for alignment and FastTree2 (Suhaimi et al. 2019). Beta diversity index calculations, along with multidimensional scaling (MDS) utilizing the Bray–Curtis dissimilarity metric, were performed using PRIMER6 and PERMANOVA+ (version 6.1.12 and version 1.0.2; Primer E, Ivybridge, UK) (Kuthoose et al. 2021). Statistical evaluations of viable counts (PERMANOVA and pairwise tests) were performed using SPSS Version 18.0 (Halim et al. 2020). Bacterial diversity was visualized employing multidimensional scaling (MDS) and 2D‐clustered column plotting. Statistical significance was defined of *p* < 0.05 (Zarkasi et al. 2018).

## 3 Results

### 3.1 Bacterial Enumeration

**FIGURE 1:**
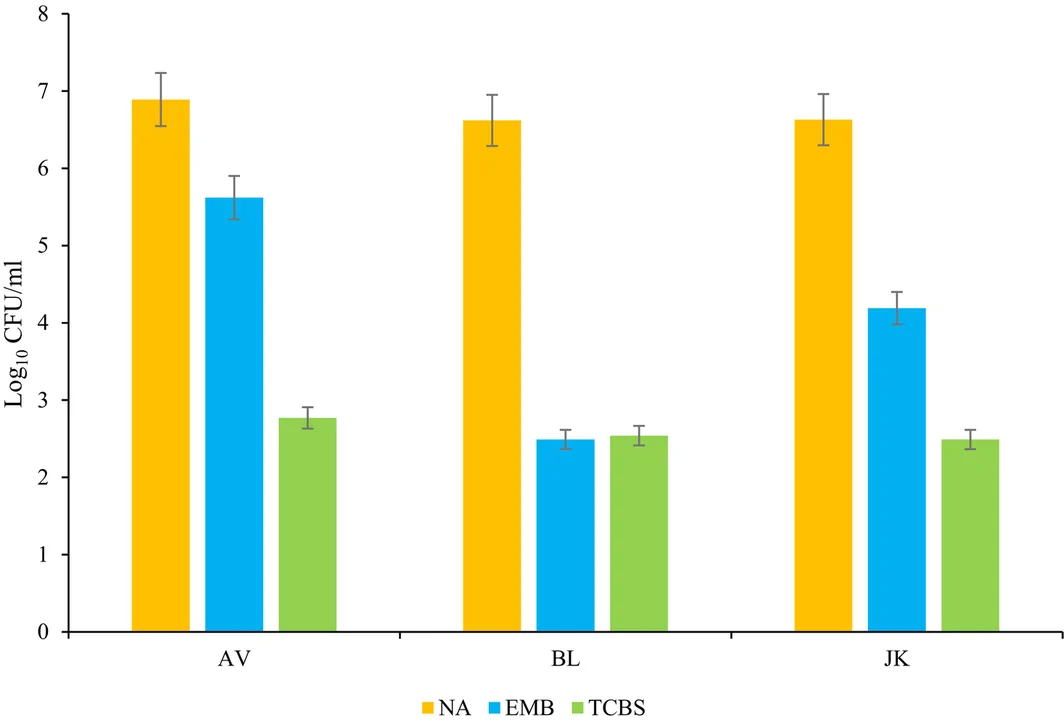
Average Total Viable Counts (TVC) expressed as Log_10_ CFU/mL of culturable bacteria from barn owl regurgitated pellets across three sampling locations. Counts were determined using nutrient agar (NA), eosin methylene blue (EMB), and thiosulphate citrate bile salts sucrose (TCBS) media. Error bars represent the standard deviation of biological replicates.

Average Total Viable Counts (TVC), expressed as Log_10_ CFU/mL, indicated that the culturable bacterial communities associated with barn owls at the AV site were more abundant compared to those at the BL and JK sites across the tested media (Figure 1). For the AV samples, average total viable counts were 6.89 Log_10_ CFU/mL on NA, 5.62 Log_10_ CFU/mL on EMB, and 2.77 Log_10_ CFU/mL on TCBS. Samples from BL recorded average total viable counts of 6.62 Log_10_ CFU/mL on NA, 2.49 Log_10_ CFU/mL on EMB, and 2.54 Log_10_ CFU/mL on TCBS. The JK samples yielded average total viable counts of 6.63 Log_10_ CFU/mL on NA, 4.19 Log_10_ CFU/mL on EMB, and 2.49 Log_10_ CFU/mL on TCBS. Comparatively, the BL site exhibited the lowest average total viable counts on NA and EMB agars, whereas the JK site showed the lowest counts on TCBS media (Figure 1).

### 3.2 Taxonomic Composition of Bacterial Communities

Metagenomic analysis generated a total of 262,707 OTUs across the three sampling locations. At the phylum level, Firmicutes constituted the most abundant taxon across all sites, accounting for 63.2% of total reads at AV, 53.2% at JK, and 28.1% at BL (Table 1). This was followed by Proteobacteria, which represented the second most abundant phylum, comprising 34.3% at AV, 45.6% at JK, and 17.7% at BL (Table 1). Notably, the phyla Actinobacteriota (26.9%) and Bacteroidota (21.9%) exhibited substantially higher relative abundance at the BL site compared to the other locations (Table 1).

**TABLE 1:** Spatial distribution and relative abundance (%) of dominant bacterial phyla associated with barn owl pellets in tropical agroecosystems.

	AV	BL	JK
	(% of total reads)		
Firmicutes	63.2	28.1	53.2
Proteobacteria	34.3	17.7	45.6
Actinobacteriota	2.5	26.9	0.4
Bacteroidota	—	21.9	0.2
Chloroflexi	—	1.3	—
Other phyla	—	4.1	—

**FIGURE 2:**
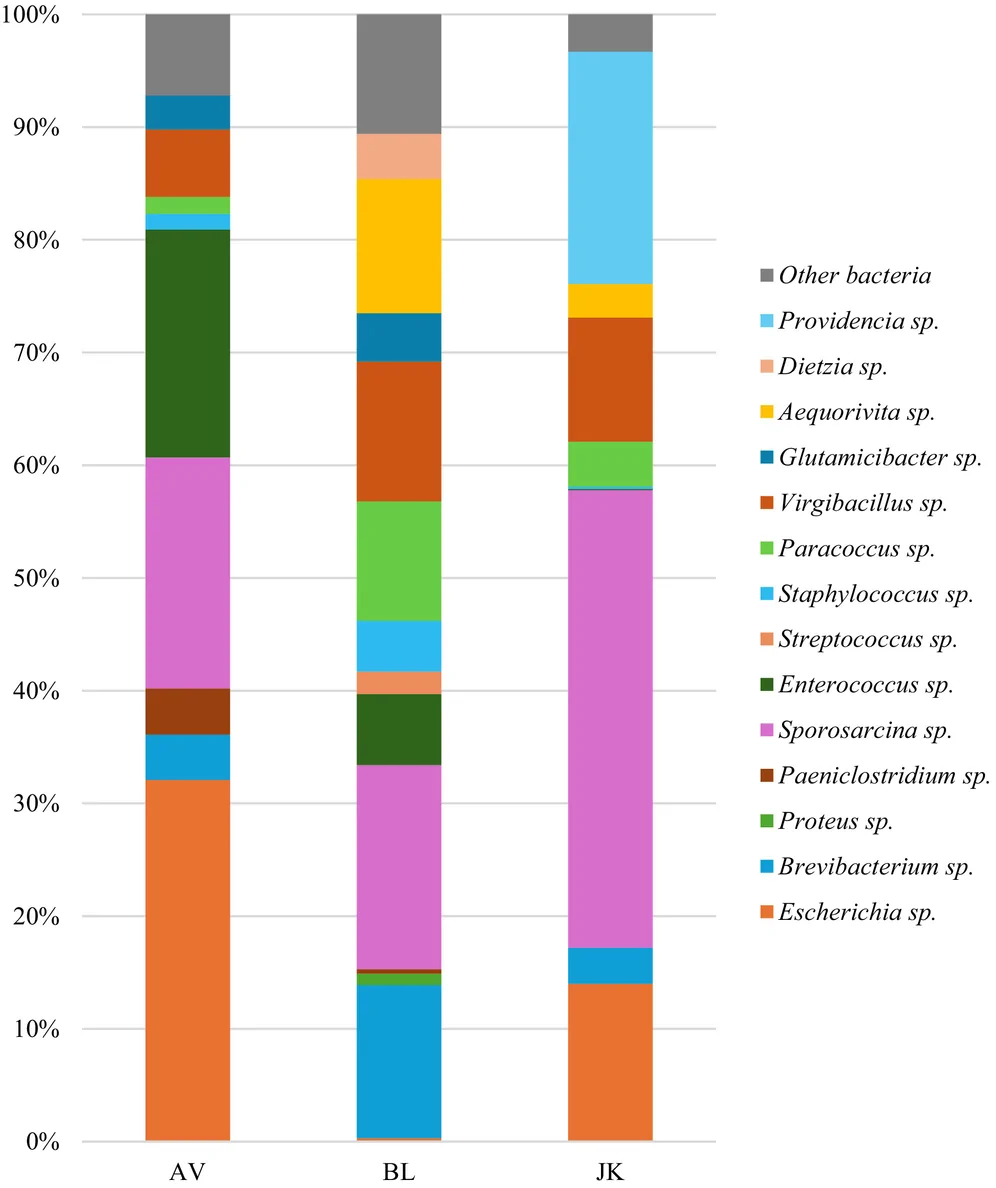
Relative abundance of major bacterial genera in barn owl regurgitated pellets across three sampling locations.

At the genus level, *Escherichia* sp. emerged as the most abundant bacterium at the AV site, representing 32.1% of total reads, followed by *Sporosarcina* sp. (20.5%) and *Enterococcus* sp. (20.2%) (Figure 2). Conversely, at the BL site, *Sporosarcina* sp. was the dominant genus (18.1%), followed by *Brevibacterium* sp. (13.6%) and *Virgibacillus* sp. (12.4%) (Figure 2). However, at the JK site, *Sporosarcina* sp. was the primary genus observed (40.6%), with *Providencia* sp. (20.6%) and *Escherichia* sp. (14.0%) following in abundance (Figure 2).

### 3.3 Bacterial Community Diversity

**FIGURE 3:**
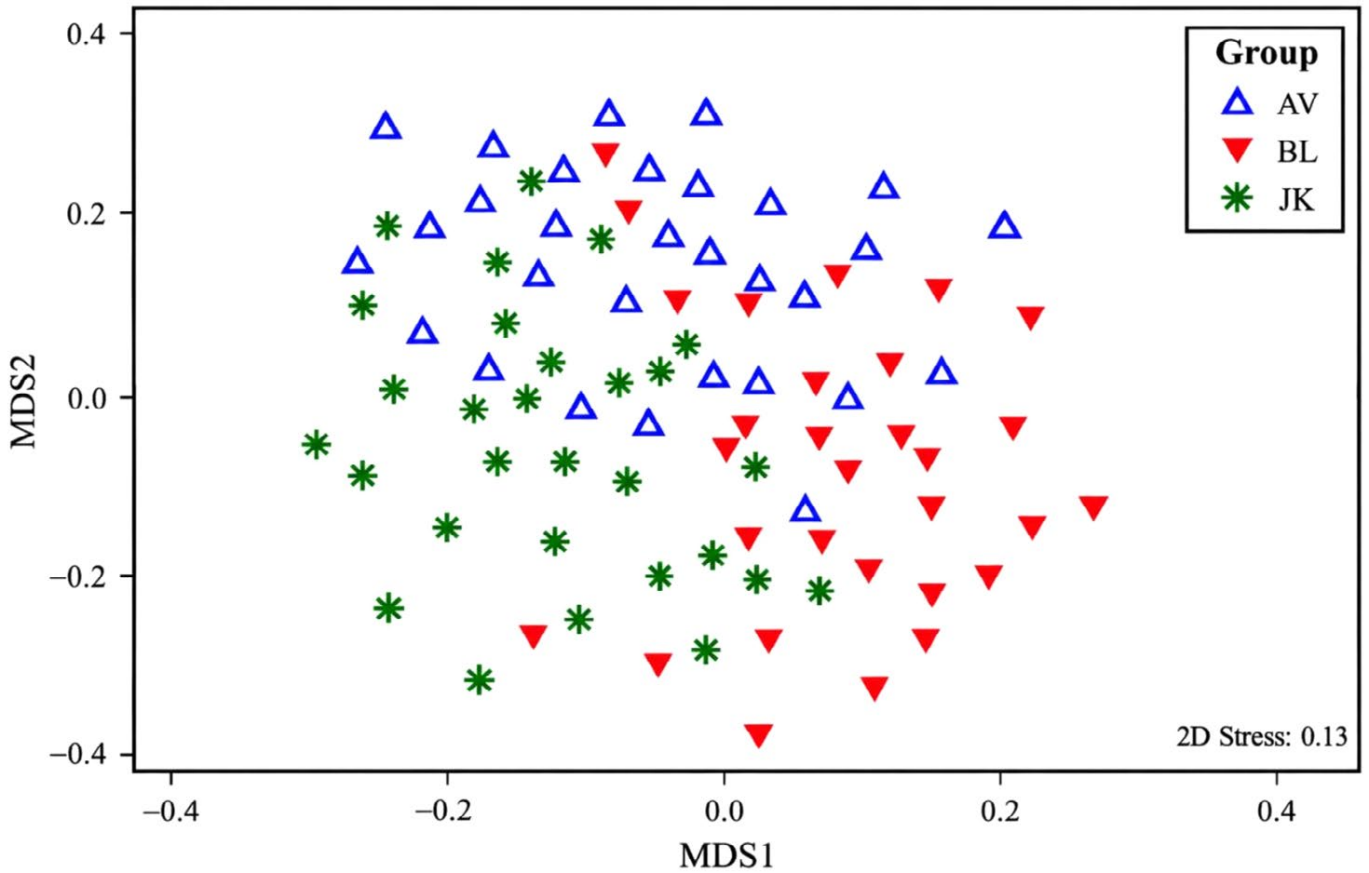
Multidimensional scaling (MDS) plot illustrating the spatial clustering of bacterial communities in barn owl regurgitated pellets across three sampling locations. Axes represent dimensionless rank‐order dissimilarities.

The distribution and diversity of the bacterial community structures varied significantly based on geographical location (Figure 3). A distinct spatial separation among the sampled populations was visually evident in the MDS plots derived from the 16S rRNA Illumina sequencing data (Figure 3). PERMANOVA results and Beta diversity analysis based on Bray‐Curtis dissimilarities indicated that differences in bacterial communities between the sampling locations were statistically significant (*F*(2, 87) = 3.14, *R* ^2^ = 0.067, *p* < 0.05) (Figure 3).

## 4 Discussion

### 4.1 Pellets as Environmental and Dietary Biosensors

The utilization of molecular approaches, particularly next‐generation sequencing has critically expanded the capacity to study bacterial communities associated with avian species (Bodawatta et al. 2018), thereby overcoming the inherent limitations of culture dependent methodologies which failed to identify uncultivated bacteria. That are overcoming the associated with avian species, thereby overcoming the inherent limitations of culture‐dependent methodologies which fail to identify uncultivable bacteria inherent limitations of culture‐dependent methodologies which fail to identify uncultivable bacteria (Johar et al. 2023; Zulfakar et al. 2023). In this study, the bacterial communities associated with the regurgitated pellets of the barn owl ( *Tyto javanica javanica* ) demonstrated significant spatial variation across three different paddy field locations. The major dominant phyla identified were Firmicutes, Proteobacteria, and Actinobacteriota that are consistent with the common bacterial flora typically found in the gastrointestinal tracts of many avian species (Bodawatta et al. 2018).

In raptorial birds, which subsist on high‐protein and high‐fat diets comprising small mammals, the gut microbiome is characteristically dominated by these taxa to facilitate the enzymatic breakdown of prey tissues and enhance metabolic efficiency (Wei et al. 2013; Kropáčková et al. 2017; Cho and Lee 2020). However, because the bacterial structure is highly dynamic and influenced by environmental conditions, diet, and geographical locations (Silvanose et al. 2001; Corl et al. 2020; Miller et al. 2020; Ahmad et al. 2021), the variations observed between the AV, BL, and JK sites suggest that regurgitated pellets act as robust biosensors reflecting both the owl's upper gastrointestinal tract and the localized agricultural environment (Garcia‐Mazcorro et al. 2021).

### 4.2 Addressing the Zoonotic Potential and *Escherichia* sp. Abundance

Previous literature frequently links wild birds to zoonotic disease transmission, noting that potentially harmful bacteria such as *Salmonella* spp., *Campylobacter* spp., and *Escherichia* sp. can be isolated from barn owls (Literák et al. 2007; Gao et al. 2021). Recent microbiome studies, however, have documented a relatively low incidence of common zoonotic pathogens in barn owl feces, casting doubt on their role as significant reservoirs for human diseases (Corl et al. 2020). While our findings align with the dominance of Firmicutes and Actinobacteriota observed in these studies, a critical divergence in our data is the high abundance of *Escherichia* sp. at specific locations (Grond et al. 2018), representing 32.1% of total reads at the AV site and 14.0% at the JK site.

This localized dominance of *Escherichia* sp. within the pellets likely stems from the owl's exposure to environmental bacteria through the ingestion of prey and frequent contact with fecal material in their habitats (Corl et al. 2020). In addition, according to an occupational health perspective, this high relative abundance of coliforms highlights a potential safety concern for agricultural workers. Farmers should exercise caution and employ proper hygiene practices during direct contact with pellets, particularly during routine nest box maintenance in the paddy fields. However, within the broader ecological context, the presence of these opportunistic and fecal‐associated bacteria in the pellets is likely a transient environmental signature acquired via their rodent diet, rather than indicative of a stable infection (Garcia‐Mazcorro et al. 2021). The detection of these bacteria in regurgitated pellets does not inherently indicate a significant threat in terms of direct zoonotic transmission but rather underscores the pellet's role in capturing the microbial footprint of the local prey, and soil before it undergoes complete digestive processing (Waite and Taylor 2014).

### 4.3 Ecological Signatures and Environmental Enrichment

Despite the presence of transient environmental coliforms, the communities were predominantly composed of normal flora and environmentally associated microorganisms (Ramezani et al. 2018; Johar et al. 2022; Sun et al. 2022). Notably, bacteria belonging to the genus *Sporosarcina* were consistently abundant across all sites, emerging as the dominant genus at JK (40.6%) and BL (18.1%), and highly prevalent at AV (20.5%). *Sporosarcina* sp. is recognized as a beneficial bacterium renowned for its role in eco‐friendly biocementation, soil enhancement, and industrial biotechnology (Lucas and Heeb 2005). While it is not considered a gut probiotic, it is environmentally valuable. The high prevalence of *Sporosarcina* sp., alongside other beneficial microbes typically associated with nitrogen fixation and biodegradation, supports the emerging paradigm that barn owls act as vectors for environmental enrichment (Feng et al. 2019; Wilcox et al. 2020; Robledo‐Mahón et al. 2022). When these regurgitated pellets degrade in terrestrial ecosystems, the associated bacterial communities are returned to the environment, thereby supporting local bacterial diversity and facilitating soil nutrient cycling (Robledo‐Mahón et al. 2022).

### 4.4 Broader Implications and Limitations

The findings of this study have substantial implications for integrated pest management strategies in agroecosystems. The data support the hypothesis that barn owls contribute more significantly to the ecological redistribution of environmental bacteria than to the spread of zoonotic diseases (Bunker et al. 2021). Therefore, their ecological function should be reconsidered to include their role as providers of microbial ecological services (García‐Fraile et al. 2015; Mokhtar et al. 2025). However, this study is limited by the inherent nature of regurgitated pellets, which serve as an indirect proxy that blends the host's upper gastrointestinal microbiota with the undigested microbial load of its prey. Future research should explore paired sampling of pellets, true fecal matter, and local soil to definitively map the directional transfer of these microbial communities within the paddy field ecosystem. (Robledo‐Mahón et al. 2022).

## 5 Conclusions

This study highlights the substantial diversity and spatial dynamics of bacterial communities associated with the regurgitated pellets of the tropical barn owl ( *Tyto javanica javanica* ) across different paddy field environments during the growing season. The microbial assemblages were predominantly characterized by beneficial and environmentally derived microorganisms, consistently highlighted by the high abundance of *Sporosarcina* sp. across all sampled locations. While potentially opportunistic bacteria such as *Escherichia* sp.

were detected in significant abundances at specific sites, their presence primarily reflects the environmental and dietary intake of the owls rather than a systemic zoonotic threat. Ultimately, the overall microbial pattern elucidated in this study supports the perspective that barn owls function as vital contributors to environmental microbial redistribution and soil enrichment, thus reinforcing their ecological value beyond their established role in agricultural rodent control.

## Author Contributions

**Kamarul Zaman Zarkasi:** conceptualization (equal), formal analysis (equal), funding acquisition (lead), project administration (lead), resources (lead), supervision (lead), validation (lead), visualization (lead), writing – original draft (equal), writing – review and editing (lead). **Mohd Hasif Ahmad Kamal:** data curation (lead), formal analysis (equal), investigation (lead), methodology (lead), writing – original draft (equal), writing – review and editing (supporting). **Muhammad Deanni Ronidean:** data curation (supporting), methodology (supporting), validation (supporting), writing – original draft (supporting), writing – review and editing (supporting). **Muhammad Syafiq Mohd Zaludin:** data curation (supporting), methodology (supporting), validation (supporting), writing – review and editing (supporting). **Mohammad Feizal Daud:** methodology (supporting), supervision (supporting), visualization (supporting), writing – original draft (supporting), writing – review and editing (supporting). **Susiana Purwantisari:** methodology (supporting), validation (supporting), writing – review and editing (supporting). **Amir Hamzah Ahmad Ghazali:** funding acquisition (supporting), supervision (supporting), validation (supporting), writing – review and editing (supporting). **Hasber Salim:** conceptualization (equal), methodology (supporting), validation (supporting), writing – original draft (supporting), writing – review and editing (supporting).

## Funding

This work is part of a project that has received funding from the Universiti Sains Malaysia via Bridging Grant (Grant number: R501‐LR‐RND003‐0000001594‐0000).

## Ethics Statement

This is an avian microbiological study that do not contact directly with the barn owl. Thus, no ethical approval is required.

## Conflicts of Interest

The authors declare no conflicts of interest.

## Data Availability

Sequences have been deposited in the NCBI under BioProject PRJNA1304600.
